# High performance of a novel point-of-care blood test for *Toxoplasma* infection in women from diverse regions of Morocco

**DOI:** 10.1080/22221751.2021.1948359

**Published:** 2021-08-22

**Authors:** Bouchra El Mansouri, Fatima Amarir, François Peyron, El Bachir Adlaoui, Raphaël Piarroux, Joseph Lykins, Majda El Abbassi, Nesma Nekkal, Nadia Bouhlal, Kamar Makkaoui, Amina Barkat, Aziza Lyaghfouri, Ying Zhou, Samira Rais, Mounia Oudghiri, Ismail Elkoraichi, Mustapha Zekri, Nezha Belkadi, Hajar Mellouk, Mohamed Rhajaoui, Allal Boutajangout, Abderrahim Sadak, Denis Limonne, Rima McLeod, Kamal El Bissati

**Affiliations:** a Institut National d’Hygiène, Rabat, Morocco; b Faculty of Sciences, Laboratory of Zoology and General Biology, Rabat, Morocco; c Laboratory of Immunity and Biodiversity, Department of Biology, Faculty of Sciences Aïn Chock, Hassan II University, Casablanca, Morocco; d Institut de Parasitologie et de Mycologie Médicale Hôpital de la Croix Rousse, Lyon, France; e LDBIO Diagnostics, Lyon, France; f Department of Internal Medicine, Virginia Commonwealth University Health System, Richmond, VA, USA; g Department of Emergency Medicine, Virginia Commonwealth University Health System, Richmond, VA, USA; h Mohamed VI Polytechnic University, CIAM, Benguerir, Morocco; i Service de Réseaux des Etablissements Santaires (SRES), Tinghir, Morocco; j The Toxoplasmosis Study Group, Obstetrics and Gynecology, Rabat, Morocco; k Research Team on Mother-Child Health and Nutrition, Faculty of Medicine & Pharmacy, Mohamed V University, Rabat, Morocco; l Service de la Protection de la Santé Infantile, Direction de la Population, Rabat, Morocco; m Department of Ophthalmology and Visual Sciences, Pediatrics (Infectious Diseases), Global Health Center, Toxoplasmosis Center, University of Chicago Medical Center, Chicago, IL, USA; n High Institute for Nursing Professions and Health Techniques (ISPITS), Casablanca, Morocco; o Department of Neurology & Physiology and Neuroscience, New York University Langone Health, New York, USA; p Institute of Molecular Engineering, University of Chicago Medical Center, Chicago, IL, USA; q Department of Biological and Forensic Sciences, Fayetteville State University, Fayetteville, NC, USA; r University Polytechnic Benguerir, CIPEM, Ben Guerir, Morocco

**Keywords:** Congenital toxoplasmosis, POC, screening, LDBIO test, diagnostics

## Abstract

Point-of-care (POC) testing for *Toxoplasma* infection has the potential to revolutionize diagnosis and management of toxoplasmosis, especially in high-risk populations in areas with significant environmental contamination and poor health infrastructure precluding appropriate follow-up and preventing access to medical care. Toxoplasmosis is a significant public health challenge in Morocco, with a relatively heavy burden of infection and, to this point, minimal investment nationally to address this infection. Herein, we analyse the performance of a novel, low-cost rapid test using fingerstick-derived whole blood from 632 women (82 of whom were pregnant) from slums, educational centres, and from nomad groups across different geographical regions (i.e. oceanic, mountainous) of Morocco. The POC test was highly sensitive and specific from all settings. In the first group of 283 women, sera were tested by Platelia ELISA IgG and IgM along with fingerstick whole blood test. Then a matrix study with 349 women was performed in which fingerstick – POC test results and serum obtained by venipuncture contemporaneously were compared. These results show high POC test performance (Sensitivity: 96.4% [IC95 90.6–98.9%]; Specificity: 99.6% [IC95 97.3–99.9%]) and high prevalence of *Toxoplasma* infection among women living in rural and mountainous areas, and in urban areas with lower educational levels. The high performance of POC test confirms that it can reduce the need for venipuncture and clinical infrastructure in a low-resource setting. It can be used to efficiently perform seroprevalence determinations in large group settings across a range of demographics, and potentially expands healthcare access, thereby preventing human suffering.

## Introduction

Congenital toxoplasmosis caused by the parasite *Toxoplasma gondii* is an emerging global health threat. The disease is a particular challenge in developing countries where health infrastructure is often lacking and access to medical care can be limited. Significant contamination of the environment with infectious oocysts in some of these countries adds to challenges [1]. Outbreaks of *Toxoplasma* infection have been recognized in many countries and caused mortality [2–5]. Contamination of a municipal reservoir water supplying Victoria, Canada, is one example of large-scale contamination of water with wild cat oocysts [6]. The cats, hosts of these parasites, are capable of shedding hundreds of millions of oocysts in their faeces over 2 weeks during primary infection. In addition, *Toxoplasma* can also be contracted by eating undercooked meat contaminated with cysts. Congenital toxoplasmosis (CT) is of particular importance because vertical transmission, when acute infection occurs during pregnancy, represents a point of intervention to reduce incidence and disease severity in affected persons. The global annual incidence of congenital toxoplasmosis is estimated to be ∼190,100 cases per year [7]. Hydrocephalus can occur, and vision loss, a source of significant morbidity, can complicate untreated, congenitally infected infants [8]. Pregnant women with acute infection often do not present any symptoms and only serologic tests confirm the infection [9]. Implementing early diagnosis and rapid initiation of treatment are key strategies in the control of congenital toxoplasmosis in France, Austria, Colombia, Brazil and other countries [10]. Treatment also includes prevention through the administration of spiramycin, which concentrates in the placenta and blocks or delays transmission of the parasite to the foetus by ∼50% [8]. If an acute infection is confirmed in pregnant women using molecular diagnostic tools, pyrimethamine, sulfadiazine, and folinic acid are the mainstays of treatment. No commercially available medicine treats the latent bradyzoite life stage. Prenatal screening and treatment have been demonstrated to be cost-effective, including in Austria and France and estimated to be so in the US [11–13]. Early identification of women who seroconvert during pregnancy becomes especially challenging in areas with limitations in health infrastructure or less robust follow-up presents a challenge to adequate management of the threat presented by *T. gondii* for human health.

It was demonstrated that the POC kit for *Toxoplasma* infection could facilitate testing with high performance in the USA (McLeod et al. manuscript in preparation). Thus, it is an alternative to address this gap with previously demonstrated strong diagnostic performance with more than 400 tests performed in the USA with a broad patient and provider acceptance [14]. Morocco, a country with high seroprevalence (∼50%) and a tradition of screening for toxoplasmosis a few times in gestation, represents a new frontier in implementing this test [15–17]. Twenty-one infants out 48,890 (3.9–8 per 10,000 live birth) were confirmed to be born with CT in different Moroccan regional hospital centres in 2015 [17]. Seventy-one percent of these were diagnosed at Rabat hospitals [17]. The establishment of a rational approach to managing congenital toxoplasmosis, especially in the developing world, was the subject of a recent study that was performed in collaboration with the National Institute of Hygiene, Rabat [17]. This concluded with the establishment/implementation of an International Collaborative POC-Prenatal Screening Program in Morocco.

We reported earlier the diagnostic performance of a lateral flow immunochromatography-based *Toxoplasma* ICT IgG–IgM (LDBIO test) using serum from venipuncture, which was highly accurate in France and the United States [18,19]. Furthermore, the whole blood variant test was found to be highly sensitive and specific [14].

Herein, we performed a national prevalence study using this novel, low-cost rapid test that is equipment free and provides results within 20 min, using whole blood obtained via finger stick for 632 women from different geographical regions (ocean and mountains) of Morocco. This was accomplished efficiently on a large scale in a short time by providing an educational session, obtaining informed consent, and collecting answers to a questionnaire assessing risk factors and demographics, together in one large group setting/session, subsequently testing was performed with blood from venipuncture and fingerstick concurrently. We found that the test is reliable, correlating well with conventional testing. With this test, we found a high prevalence of *Toxoplasma* infection in rural areas and women with low educational levels. The specificity and the sensitivity of the test were very high in all sites when compared to conventional testing for IgG and IgM from Bio-Rad ELISA test. Matrix study comparison between the results of whole blood obtained by fingerstick and 15 μL of serum obtained from venipuncture showed high sensitivity: (96.4%) with 4 false negatives and 1 false positive. These results have implications for potentially reducing the need for venipuncture and mitigating the lack of healthcare infrastructure with technical sophistication at the site for processing samples, especially in geographical areas with barriers to access for populations with a high burden of infection.

## Materials and methods

### Study area

The study was performed in Casablanca, Rabat and Aït Hani (Tinghir). In Casablanca, data were collected from three locations: (1) Communal Hospital Center Al Waha, Sidi Moumen, (2) Higher Institute of Nursing Professions and Health Technologies (ISPITS), and (3) Faculty of Sciences Aïn Chock, University Hassan II (FSAC).

### Human subjects’ involvement and characteristics

The 632 participants recruited in these centres were female, ages 17–70 years old. Among them, 82 were pregnant women. The distribution of these participants in the three regions is as follows: *Casablanca region*: 36 underserved women patients from the poor neighbourhoods of Sidi Moumen, 188 female students from ISPITS and 103 female students from FSAC; *Rabat region:* 184 women presented to the diagnostic centre at the National Institute of Hygiene in Rabat; *Aït Hani region:* 121 nomad women living in the mountains near Tinghir. Demographics including age, socioeconomic status (as described by living area, professional activity, education level) and clinical/risk factor data (contamination mode, consumption of undercooked meat, raw vegetables, and unfiltered water, cat contact, soil contact, gestational history in case of pregnant women) were collected from participants during their visits to these centres. Two blood samples were collected from each participant, one via fingerstick and the other via venipuncture.

### Matrix study

Participants provided whole blood via fingerstick. A lancet and capillary tube was used to collect ∼30 µL of blood (half a 60 µL capillarity tube, by visual estimate), which was placed on the sample collection pad of the LDBIO test kit, followed by 4 drops of buffer provided in the kit. Twenty minutes later, the results were interpreted independently by two independent observers and photographed for later comparison to the serological status of the subjects. In parallel, participants had blood obtained by venipuncture and serum obtained from this sample after centrifugation. 15 µL of serum were placed on a new LDBIO kit and the results were compared to the whole blood POC test result and the serum from the venipuncture sample.

### Reference standard test

All sera were transported to the *Toxoplasma* Serology Laboratory at the National Institute of Hygiene (Health Ministry) for ELISA Platelia™ Toxo IgG: ref 72840 and Platelia™ Toxo IgM: ref 72841 assays (BIO-RAD, Marnes la Coquette, France). The ELISA test was used as a reference standard for comparison to LDBIO tests for each patient. Additionally, the sensitivity and specificity of the LDBIO test were determined.

### Reference Western blot test

In addition to ELISA Bio-Rad test, we used Western blot TOXO II as a reference test for comparing the efficacy of LDBIO test. Briefly, anonymized samples from the matrix study were also sent to LDBIO Diagnostics (Lyon, France), where Toxo II WB IgG test (LDBIO Diagnostics, Lyon France), recognized as equivalent to the reference gold standard tests IgG Dye test in France [20], was performed on all samples. The test was performed blindly in samples according to the manufacturer’s instruction. Briefly, 10 µl of sample was used and incubated with the test strip in different test buffers; dilution buffer (for 90 min), conjugate antibodies (for 60min), and substrate (for 60 min). The test was considered positive if at least 3 bands, among P30, P31, P33, P40, or P45, were visible in the strip, including P30. Band positioning and test validation were performed by comparison to positive control included in the kit.

### Ethics

All participants provided written informed consent. The study was conducted with ethical standards for human experimentation established in the Declaration of Helsinki, with prior institutional review approval from National Institute of Hygiene and Moroccan Ministry of Health. The privacy and safety of the participants were ensured, and informed consent obtained from participants was stored and kept confidential at the National Institute of Hygiene. Biological samples were collected under the norms and standards established by the Moroccan Ministry of Health.

### Statistical analysis

Sensitivity and specificity were obtained using PROC FREQ and approximate confidence intervals using Wilson’s method with correction of continuity were reported. All *p*-values reported were based on chi-square tests. Comparisons between sites were made using a chi-square test. All statistical analyses were performed using Stata software, version 13.

## Results

### Study design and samples subjects

**Figure 1.**
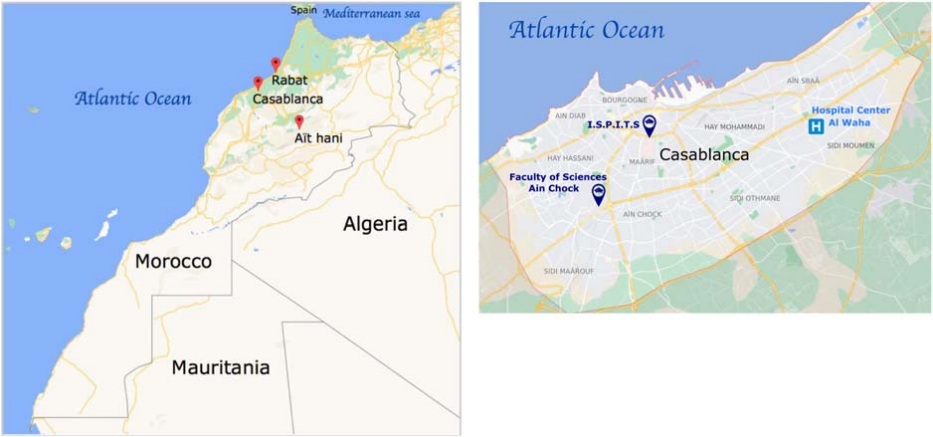
(a), Map of Morocco with the areas of Casablanca, Rabat and Ait Hani. (b), sites in Casablanca region (ISPITS, FSAC, and Al Waha Center).

The 632 participants in this study are from rural and urban areas of 3 diverse geographical sites of Morocco (Casablanca, Rabat and Ait Hani) (Figure 1).

### Comparison of rapid test with conventional serologic testing

Sera from patients were processed and analysed for IgG and IgM using ELISA from Bio-Rad. Data are reported in Table 1 and Table 1S (Confidence intervals are within brackets).

**Table 1.** Comparison of rapid test with conventional serologic testing: sera from patients were processed and analysed for IgG and IgM using ELISA from Bio-Rad (confidence intervals are within brackets).

Region	Location	Positive	Negative	Positivity rate	Sensitivity	Specificity
Rabat	Rabat	102	82	55% [47.9–62.7%]	96% [89.7–98.7]	100% [94.4–100]
Tinghir	Aït Hani	57	64	47% [38.0–56.4%]	93% [82.2–97.7]	98% [90.4–99.9]
Casablanca	Al Waha	16	20	44% [28.3–61.7]	94% [67.7–99.7]	100%[79.9–100]
	ISPTS	33	155	18% [12.5–23.9]	100% [87.0–100]	100% [97.0–100]
	FSAC	18	85	17% [11.0–26.5]	100% [78.1–100]	100% [94.6–100]
	Total	226	406	36% [32.0–39.7]	97% [93.5–98.6]	100% [98.4–100]

### Risk factors on the epidemiology of toxoplasmosis among patients

**Figure 2.**
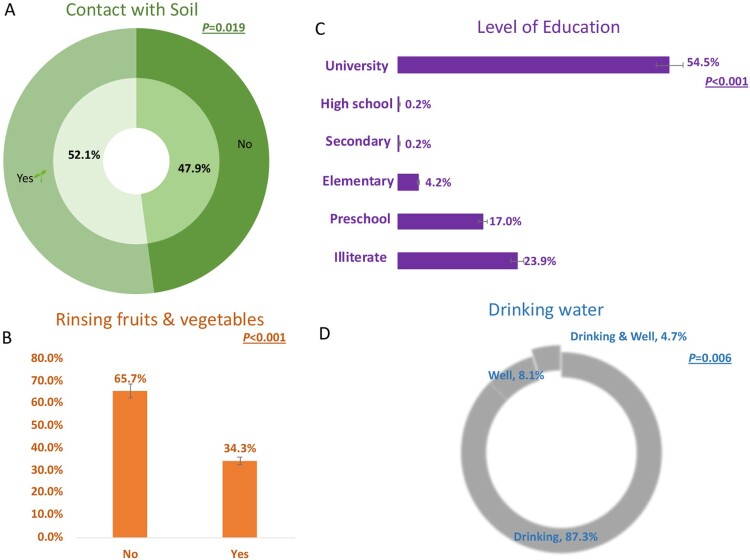
Risk factors and serological test results. There is a correlation between the results of the percent positivity of LDBIO test and contact with soil (A), absence of washing fruits & vegetables before use (B), low educational level (C), and drinking water from wells (D).

We performed a questionnaire focusing on knowledge of toxoplasmosis in all study sites. We found that the majority of patients acknowledged the risk factors of toxoplasmosis through the contact of soil, unwashed fruits and vegetables, drinking water from the supply system, or wells (Figure 2). Additionally, the level of education is a risk factor for infection with *T. gondii*. There was a high prevalence of *Toxoplasma* infection among women living in rural and mountainous areas, and in urban areas with lower educational levels. Other risk factors included exposure to soil, well water and not washing fruits and vegetables prior to consuming them (Figure 2).

### Matrix study

**Figure 3.**
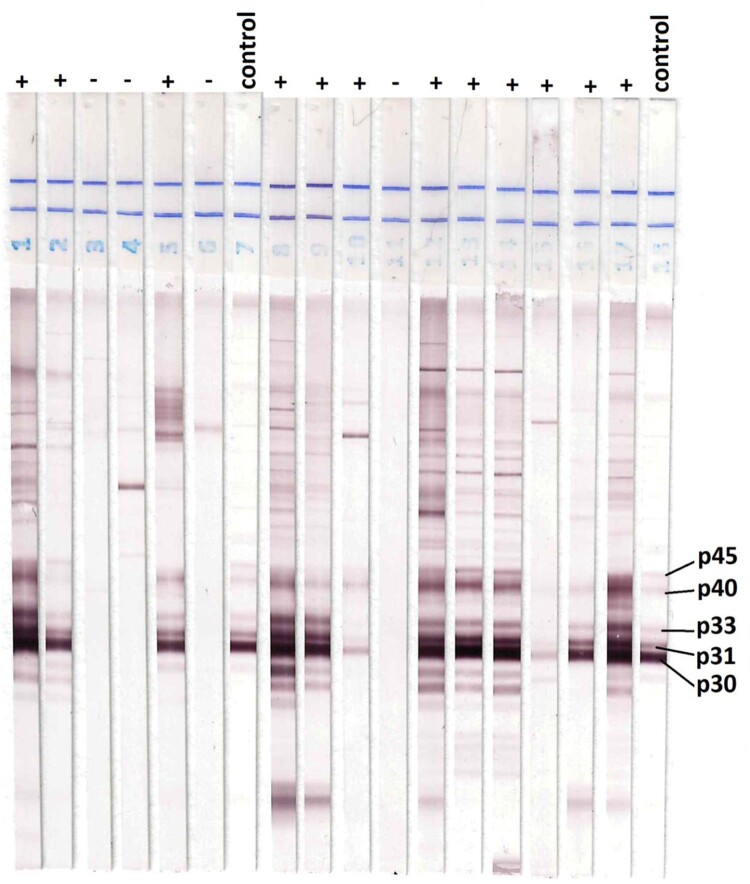
Western blot results of samples from Rabat. The comparison of additional bands (P31, P33, P40, P45, and particularly P30) on the strip of IgG indicates infection with *Toxoplasma*.

We performed a matrix study by comparing POC test from 30 µL of whole blood fingerstick compared to 15 µL of serum obtained by venipuncture from the same patient and analysed on the same day. The results were compared to the Western Blot (WB) LDBIO TOXO II test used as another reference standard (Table 2). We found a high performance of the LDBIO Kit with a sensitivity of 96.4% [IC95 90.6–98.9%] and a specificity of 99.6% [IC95 97.3–99.9%]. The correlation of the test on blood vs serum on 349 people was high, with very little difference between the two types of samples; 4 false negatives and 1 false positive were detected. This difference compared to the other reference ELISA Bio-Rad test is negligible (Chi^2^, Sensitivity *p = *0.99; Specificity *p = *0.7) (Figure 3).

**Table 2.** Comparison of POC whole blood fingerstick with POC serum.

	Results of LDBIO Kit whole blood (30 µL) fingerstick VS LDBIO Kit (15 µL) of serum obtained by venipuncture		
	POC Whole blood test	POC Serum test	Western Blot TOXO II test
Positive	108	112	112
Negative	236	237	237
	5 discordant (1 false positive and 4 false negatives)		Total: 349
Performance	Sensitivity: 96.4% [IC95 90.6–98.9%] Specificity: 99.6% [IC95 97.3–99.9%]		

### LDBIO kit for pregnant women

**Figure 4.**
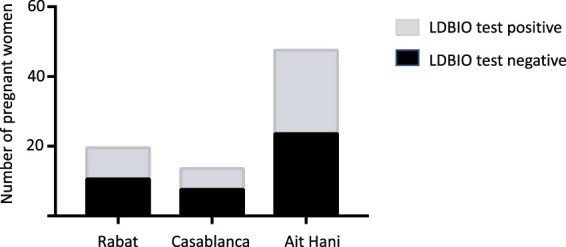
Number of pregnant women who had a negative or positive result with the LDBIO kit.

We determined the number of pregnant women enrolled in this study and the presence of *Toxoplasma* antibodies in 3 locations. Data are presented in Figure 4. We correlated the response of LDBIO whole blood test to ELISA tests using Bio-Rad assays and determined the sensitivity and specificity of the LDBIO test in this subpopulation. From a total of 82 pregnant women, 38 were confirmed positive and 44 negative, with only 2 discordant. Additionally, there is no significant difference (Sensitivity, *p =* 0.61; Specificity *p = *0.74) in comparison with the general population.

## Discussion

Herein, the ICT IgG–IgM (LDBIO test) for whole blood has proven to be effective in identifying patients with anti-*Toxoplasma* antibodies in Morocco. The sensitivity and the specificity are very high in all study sites.

Casablanca and Rabat are situated along the Atlantic Ocean and located in the west of Morocco while Tinghir, is located in the south of the High Atlas Mountains. Sidi Moumen is an arrondissement and northeastern suburb of Casablanca. It contains shantytowns and slums, and the majority of the women utilizing the community hospital has comparatively low educational levels, and therefore potentially lack awareness of the disease. In contrast, students or health professionals from ISPITS and FSAC might be more likely to have some knowledge of the disease. Rabat, and in particular the National Institute of Hygiene, is another study site, where volunteers were enrolled. The other primary study site was centre Aït Hani in Tinghir, a city centred on an oasis and composed of many smaller villages. It is located at 2000 m above sea level, south of the High Atlas Mountains. Nomads are the predominant ethnic group comprising this city’s population, with many living and working in agriculture and trade.

The POC test from areas of Casablanca (ISPITS and FSAC) showed a seroprevalence of ∼18%, with patients composed of college students, professors, and healthcare professionals. This was lower than rates reported in the literature in different cities of Morocco (e.g. Rabat 50.6% [15], Essaouira 45% [21], Kenitra 37.7%, Tetouan 42% [21]) and neighbouring countries (Algeria 47.8% [22] or Tunisia 47.7% [23]). However, patients from the same region (Casablanca) but from poor neighbourhoods (Al Waha Sidi Moumen) with many of them are illiterate, were much more likely to test positive for antibodies against the parasite. The seroprevalence was ∼44%, similar to levels reported in the literature. As expected, the same prevalence (47.1%) was seen in nomads at Aït Hani with the same association with lower socioeconomic status. This represents, to our knowledge, the first assessment of seroprevalence in this population, likely due to difficulties establishing contact with this population. These differences in seroprevalence potentially suggest that the patients in those areas are unaware of the risk of toxoplasmosis. Thus, women who participate in educational programs centred on risk factors and disease prevention have the potential to reduce exposure to the parasite and, thereby, disease burden. Anecdotally, we encountered many cases of recurrent pregnancy loss, especially in the nomadic peoples (data not shown). These women were never tested for toxoplasmosis or other congenital diseases during their first trimester and likely did not receive adequate follow-up/prenatal care. Water contamination, contact with the soil, and living with domestic animals might explain the high prevalence of *T. gondii* infection within this population.

After testing the initial participants, we added testing with western blot for the remaining participants. We performed a matrix study with 349 women by comparing POC from whole blood via finger stick and serum obtained by venipuncture on the same day. The results, when compared to western blot reference test measuring *Toxoplasma* IgG antibodies, show high performance of the LDBIO kit, (Sensitivity: 96.4% [IC95 90.6–98.9%], Specificity: 99.6% [IC95 97.3–99.9%]), with 4 false negatives and 1 false positive. Like in France and the United States, the results presented herein confirm the strong diagnostic performance of this test in a newly studied population. The global picture of the genotype of the parasites in Moroccan patients is still lacking. Only one study determined the genotypes of *T. gondii* occurring in Morocco as type III genotype [24]. Studies have shown that the parasitic strains in South America are more virulent than those in France and US [25]. US patients are infected with type II, I/III, I/IIIa, and atypical parasites, and French patients predominantly with type II strains. This suggests that the test performs well in diverse populations of hosts and parasites, and the data presented herein confirm the tests’ strong performance for genotypes and participants present in Morocco.

This current study made use of novel, well-validated POC blood testing, and has numerous potential “spillover” benefits for other causes of maternal/child morbidity and mortality in the country [11–13]. This approach supports the establishment of a monthly screening program for the acquisition of *Toxoplasma* infection during gestation with the intent of improving maternal and child health [26]. This has the potential to be especially important for the care of the nomadic peoples of Morocco with whom it has been previously difficult to establish contact. This study is the first, to our knowledge, to study the performance of POC testing for toxoplasmosis in different populations from within Morocco. Further, it provides proof of concept that POC-based screening might be feasible for other congenital infections. Recently, many countries invested in the implementation of guidelines to face public health challenges due to toxoplasmosis [27]. Examples included the congenital toxoplasmosis centres in Brazil, the Center at the Universidad del Quindio in Colombia [28]. After the implementation of these guidelines for helping pregnant women during their first trimester and with monthly testing, there was a significant reduction in the percentage of children seen with severe neurological damage in Quindio [17].

With substantial numbers of infants born with hydrocephalus due to congenital toxoplasmosis reported in regional hospital centres in Morocco [17], introducing this kit for prenatal and neonatal screening will be of potential value. It was already shown that prenatal screening and treatment of infected women reduce the risk of transmission of the parasite and sequelae in infants [10,17]. Different countries like France, Austria, Brazil, Slovenia, Uruguay, and Panama have prioritized their investments to address congenital toxoplasmosis by introducing screening. Some new measures are taken recently by the government of Morocco plan for the introduction of the POC toxoplasmosis test in 2023. Combining the *Toxoplasma* test in a multiplex test with other treatable or preventable congenital diseases, including rubella, CMV, hepatitis B, syphilis, and HIV, will likely further enhance screening programs in the country. As discussed above, these measures will save lives, prevent human suffering, and provide spillover benefits for maternal and child health.

Our current study in Morocco represents a step forward towards the implementation of POC diagnostics in toxoplasmosis for pregnant women. The lateral flow immunochromatography LDBIO kit detects both *Toxoplasma* IgG and IgM antibodies in serum and blood (as occurs during early seroconversion after acute infection). We demonstrate that among the 82 pregnant women enrolled in our study, only 2 were found to be discordant when compared to the reference test. These results hold promise given the rapid turnaround time, simplicity, and low cost. The LDBIO test can be introduced easily in the population living in mountains (i.e. Aït Hani location) at this remote site. There is difficulty with access to health care. Additionally, it could be used for mass surveillance and implemented for a POC test-based prenatal screening. Despite the need for confirmatory screening using reference tests for pregnant women testing positive during early gestation, the test is cost-saving. While the LDBIO test has strong diagnostic performance, it does not distinguish between acute and chronic infection, as it tests for the presence of both IgG and IgM against *T. gondii*. A positive POC test prior to 12 weeks of gestation requires further confirmatory testing and guidance from the physicians. An additional testing for avidity of antibody is required for those who have *T. gondii*-specific IgM.

The integration of LDBIO into a national POC test program for the prevention of acquisition of *Toxoplasma* by pregnant women who test negative prior to pregnancy has the potential to be of great utility, as these women are at risk of transmitting the infection to their fetus for the rest of their pregnancy. It is estimated that the cost of the LDBIO test that recognizes both IgG and IgM is $5–8. This is cheaper than the conventional test for IgG and IgM, which costs in total $16 (paid in France by the National Welfare System) or $35–$650 (paid in US), without taking into consideration the fees imposed by the hospital or healthcare facilities. These include training of healthcare personnel involved in venipuncture, sample processing, costs of required infrastructure, machine calibration, etc. Similar costs are not found in a system relying on POC testing. If the cost is $16–35 for testing once, then the total cost for a pregnant woman will be $128–280 (8 tests needed for testing across gestation; 7 tests during pregnancy and 1 test after delivery). However, $40–64 for 8 LDBIO tests is more affordable and provides the opportunity for a pregnant woman to control her own health care, facilitating early diagnosis and treatment of this infection, reducing neonatal morbidity and mortality.

## Conclusion

In conclusion, we demonstrated that the LDBIO kit test for *Toxoplasma* antibodies showed high accuracy and performance in low and high-risk populations from 3 geographical regions of Morocco. These results can help all pregnant women but are especially important for populations at high risk of infection and those living in mountain areas, affording access to a POC-based prenatal screening program that mitigates most of the cost of serological testing and movement to healthcare facilities.

## Supplementary Material

Table_2S.docx

Table_1S.docx
