# Supplementary material for: High performance of a novel point-of-care blood test for Toxoplasma infection in women from diverse regions of Morocco
Source: Emerg Microbes Infect. 2021 Aug 22;10(1):1675–82. doi: 10.1080/22221751.2021.1948359 (PMC8381951; doi:10.1080/22221751.2021.1948359)
Supplement: Table_2S.docx [file TEMI_A_1948359_SM9341.docx]

**Supporting information**

**Table 2S**

| **Participant Number** | **Area  of study** | **Age** | **POC blood test** | **POC  serum test** | **WB Toxo II IgG** | **DISCORDANCE POC BLOOD/POC SERUM** | **DISCORDANCE POC BLOOD/WB** | **DISCORDANCE POC SERUM/WB** |
| --- | --- | --- | --- | --- | --- | --- | --- | --- |
| 1 | Casablanca^1^ | 20 | NEG | NEG | NEG | Concordance | Concordance | Concordance |
| 2 | Casablanca^1^ | 20 | NEG | NEG | NEG | Concordance | Concordance | Concordance |
| 3 | Casablanca^1^ | 20 | NEG | NEG | NEG | Concordance | Concordance | Concordance |
| 4 | Casablanca^1^ | 21 | NEG | NEG | NEG | Concordance | Concordance | Concordance |
| 5 | Casablanca^1^ | 20 | NEG | NEG | NEG | Concordance | Concordance | Concordance |
| 6 | Casablanca^1^ | 20 | NEG | NEG | NEG | Concordance | Concordance | Concordance |
| 7 | Casablanca^1^ | 22 | POS | POS | POS | Concordance | Concordance | Concordance |
| 8 | Casablanca^1^ | 20 | NEG | NEG | NEG | Concordance | Concordance | Concordance |
| 9 | Casablanca^1^ | 20 | NEG | NEG | NEG | Concordance | Concordance | Concordance |
| 10 | Casablanca^1^ | 20 | NEG | NEG | NEG | Concordance | Concordance | Concordance |
| 11 | Casablanca^1^ | 20 | NEG | NEG | NEG | Concordance | Concordance | Concordance |
| 12 | Casablanca^1^ | 19 | NEG | NEG | NEG | Concordance | Concordance | Concordance |
| 13 | Casablanca^1^ | 19 | NEG | NEG | NEG | Concordance | Concordance | Concordance |
| 14 | Casablanca^1^ | 20 | NEG | NEG | NEG | Concordance | Concordance | Concordance |
| 15 | Casablanca^1^ | 18 | NEG | NEG | NEG | Concordance | Concordance | Concordance |
| 16 | Casablanca^1^ | 20 | POS | POS | POS | Concordance | Concordance | Concordance |
| 17 | Casablanca^1^ | 19 | NEG | NEG | NEG | Concordance | Concordance | Concordance |
| 18 | Casablanca^1^ | 18 | NEG | NEG | NEG | Concordance | Concordance | Concordance |
| 19 | Casablanca^1^ | 19 | NEG | NEG | NEG | Concordance | Concordance | Concordance |
| 20 | Casablanca^1^ | 21 | NEG | NEG | NEG | Concordance | Concordance | Concordance |
| 21 | Casablanca^1^ | 20 | NEG | NEG | NEG | Concordance | Concordance | Concordance |
| 22 | Casablanca^1^ | 20 | NEG | NEG | NEG | Concordance | Concordance | Concordance |
| 23 | Casablanca^1^ | 19 | NEG | NEG | NEG | Concordance | Concordance | Concordance |
| 24 | Casablanca^1^ | 21 | NEG | NEG | NEG | Concordance | Concordance | Concordance |
| 25 | Casablanca^1^ | 19 | POS | POS | POS | Concordance | Concordance | Concordance |
| 26 | Casablanca^1^ | 19 | NEG | NEG | NEG | Concordance | Concordance | Concordance |
| 27 | Casablanca^1^ | 19 | NEG | NEG | NEG | Concordance | Concordance | Concordance |
| 28 | Casablanca^1^ | 19 | NEG | NEG | NEG | Concordance | Concordance | Concordance |
| 29 | Casablanca^1^ | 19 | NEG | NEG | NEG | Concordance | Concordance | Concordance |
| 30 | Casablanca^1^ | 19 | NEG | NEG | NEG | Concordance | Concordance | Concordance |
| 31 | Casablanca^1^ | 18 | NEG | NEG | NEG | Concordance | Concordance | Concordance |
| 32 | Casablanca^1^ | 19 | NEG | NEG | NEG | Concordance | Concordance | Concordance |
| 33 | Casablanca^1^ | 19 | NEG | NEG | NEG | Concordance | Concordance | Concordance |
| 34 | Casablanca^1^ | 20 | NEG | NEG | NEG | Concordance | Concordance | Concordance |
| 35 | Casablanca^1^ | 19 | NEG | NEG | NEG | Concordance | Concordance | Concordance |
| 36 | Casablanca^1^ | 20 | NEG | NEG | NEG | Concordance | Concordance | Concordance |
| 37 | Casablanca^1^ | 18 | NEG | NEG | NEG | Concordance | Concordance | Concordance |
| 38 | Casablanca^1^ | 19 | NEG | NEG | NEG | Concordance | Concordance | Concordance |
| 39 | Casablanca^1^ | 20 | NEG | NEG | NEG | Concordance | Concordance | Concordance |
| 40 | Casablanca^1^ | 20 | NEG | NEG | NEG | Concordance | Concordance | Concordance |
| 41 | Casablanca^1^ | 20 | NEG | NEG | NEG | Concordance | Concordance | Concordance |
| 42 | Casablanca^1^ | 19 | NEG | NEG | NEG | Concordance | Concordance | Concordance |
| 43 | Casablanca^1^ | 20 | NEG | NEG | NEG | Concordance | Concordance | Concordance |
| 44 | Casablanca^1^ | 21 | NEG | NEG | NEG | Concordance | Concordance | Concordance |
| 45 | Casablanca^1^ | 19 | NEG | NEG | NEG | Concordance | Concordance | Concordance |
| 46 | Casablanca^1^ | 20 | NEG | NEG | NEG | Concordance | Concordance | Concordance |
| 47 | Casablanca^1^ | 20 | NEG | NEG | NEG | Concordance | Concordance | Concordance |
| 48 | Casablanca^1^ | 19 | NEG | NEG | NEG | Concordance | Concordance | Concordance |
| 49 | Casablanca^1^ | 20 | NEG | NEG | NEG | Concordance | Concordance | Concordance |
| 50 | Casablanca^1^ | 20 | NEG | NEG | NEG | Concordance | Concordance | Concordance |
| 51 | Casablanca^1^ | 20 | NEG | NEG | NEG | Concordance | Concordance | Concordance |
| 52 | Casablanca^1^ | 20 | POS | POS | POS | Concordance | Concordance | Concordance |
| 53 | Casablanca^1^ | 20 | NEG | NEG | NEG | Concordance | Concordance | Concordance |
| 54 | Casablanca^1^ | 21 | NEG | NEG | NEG | Concordance | Concordance | Concordance |
| 55 | Casablanca^1^ | 19 | NEG | NEG | NEG | Concordance | Concordance | Concordance |
| 56 | Casablanca^1^ | 20 | POS | POS | POS | Concordance | Concordance | Concordance |
| 57 | Casablanca^1^ | 20 | NEG | NEG | NEG | Concordance | Concordance | Concordance |
| 58 | Casablanca^1^ | 24 | POS | POS | POS | Concordance | Concordance | Concordance |
| 59 | Casablanca^1^ | 20 | POS | POS | POS | Concordance | Concordance | Concordance |
| 60 | Casablanca^1^ | 20 | NEG | NEG | NEG | Concordance | Concordance | Concordance |
| 61 | Casablanca^1^ | 19 | NEG | NEG | NEG | Concordance | Concordance | Concordance |
| 62 | Casablanca^1^ | 19 | NEG | NEG | NEG | Concordance | Concordance | Concordance |
| 63 | Casablanca^1^ | 20 | NEG | NEG | NEG | Concordance | Concordance | Concordance |
| 64 | Casablanca^1^ | 20 | NEG | NEG | NEG | Concordance | Concordance | Concordance |
| 65 | Casablanca^1^ | 19 | POS | POS | POS | Concordance | Concordance | Concordance |
| 66 | Casablanca^1^ | 19 | NEG | NEG | NEG | Concordance | Concordance | Concordance |
| 67 | Casablanca^1^ | 19 | NEG | NEG | NEG | Concordance | Concordance | Concordance |
| 68 | Casablanca^1^ | 19 | NEG | NEG | NEG | Concordance | Concordance | Concordance |
| 69 | Casablanca^1^ | 20 | NEG | NEG | NEG | Concordance | Concordance | Concordance |
| 70 | Casablanca^1^ | 19 | NEG | NEG | NEG | Concordance | Concordance | Concordance |
| 71 | Casablanca^1^ | 18 | POS | POS | POS | Concordance | Concordance | Concordance |
| 72 | Casablanca^1^ | 19 | NEG | NEG | NEG | Concordance | Concordance | Concordance |
| 73 | Casablanca^1^ | 19 | NEG | NEG | NEG | Concordance | Concordance | Concordance |
| 74 | Casablanca^1^ | 20 | NEG | NEG | NEG | Concordance | Concordance | Concordance |
| 75 | Casablanca^1^ | 21 | NEG | NEG | NEG | Concordance | Concordance | Concordance |
| 76 | Casablanca^1^ | 19 | NEG | NEG | NEG | Concordance | Concordance | Concordance |
| 77 | Casablanca^1^ | 19 | POS | POS | POS | Concordance | Concordance | Concordance |
| 78 | Casablanca^1^ | 19 | NEG | NEG | NEG | Concordance | Concordance | Concordance |
| 79 | Casablanca^1^ | 19 | NEG | NEG | NEG | Concordance | Concordance | Concordance |
| 80 | Casablanca^1^ | 20 | POS | POS | POS | Concordance | Concordance | Concordance |
| 81 | Casablanca^1^ | 19 | POS | POS | POS | Concordance | Concordance | Concordance |
| 82 | Casablanca^1^ | 19 | NEG | NEG | NEG | Concordance | Concordance | Concordance |
| 83 | Casablanca^1^ | 39 | POS | POS | POS | Concordance | Concordance | Concordance |
| 84 | Casablanca^1^ | 19 | NEG | NEG | NEG | Concordance | Concordance | Concordance |
| 85 | Casablanca^1^ | 21 | POS | POS | POS | Concordance | Concordance | Concordance |
| 86 | Casablanca^1^ | 18 | NEG | NEG | NEG | Concordance | Concordance | Concordance |
| 87 | Casablanca^1^ | 19 | NEG | NEG | NEG | Concordance | Concordance | Concordance |
| 88 | Casablanca^1^ | 19 | NEG | NEG | NEG | Concordance | Concordance | Concordance |
| 89 | Casablanca^1^ | 18 | NEG | NEG | NEG | Concordance | Concordance | Concordance |
| 90 | Casablanca^1^ | 19 | NEG | NEG | NEG | Concordance | Concordance | Concordance |
| 91 | Casablanca^1^ | 20 | NEG | NEG | NEG | Concordance | Concordance | Concordance |
| 92 | Casablanca^1^ | 19 | NEG | NEG | NEG | Concordance | Concordance | Concordance |
| 93 | Casablanca^1^ | 18 | NEG | NEG | NEG | Concordance | Concordance | Concordance |
| 94 | Casablanca^1^ | 19 | NEG | NEG | NEG | Concordance | Concordance | Concordance |
| 95 | Casablanca^1^ | 18 | NEG | NEG | NEG | Concordance | Concordance | Concordance |
| 96 | Casablanca^1^ | 18 | NEG | NEG | NEG | Concordance | Concordance | Concordance |
| 97 | Casablanca^1^ | 18 | NEG | NEG | NEG | Concordance | Concordance | Concordance |
| 98 | Casablanca^1^ | 19 | NEG | NEG | NEG | Concordance | Concordance | Concordance |
| 99 | Casablanca^1^ | 19 | NEG | NEG | NEG | Concordance | Concordance | Concordance |
| 100 | Casablanca^1^ | 19 | NEG | NEG | NEG | Concordance | Concordance | Concordance |
| 101 | Casablanca^1^ | 21 | NEG | NEG | NEG | Concordance | Concordance | Concordance |
| 102 | Casablanca^1^ | 22 | NEG | NEG | NEG | Concordance | Concordance | Concordance |
| 103 | Casablanca^1^ | 20 | POS | POS | POS | Concordance | Concordance | Concordance |
| 104 | Casablanca^1^ | 20 | NEG | NEG | NEG | Concordance | Concordance | Concordance |
| A50 | Casablanca^1^ | 20 | NEG | NEG | NEG | Concordance | Concordance | Concordance |
| A51 | Casablanca^1^ | 19 | POS | POS | POS | Concordance | Concordance | Concordance |
| A52 | Casablanca^1^ | 19 | POS | POS | POS | Concordance | Concordance | Concordance |
| A53 | Casablanca^1^ | 19 | NEG | NEG | NEG | Concordance | Concordance | Concordance |
| A55 | Casablanca^1^ | 18 | POS | POS | POS | Concordance | Concordance | Concordance |
| A61 | Casablanca^1^ | 19 | NEG | NEG | NEG | Concordance | Concordance | Concordance |
| A62 | Casablanca^1^ | 20 | NEG | NEG | NEG | Concordance | Concordance | Concordance |
| A64 | Casablanca^1^ | 21 | NEG | NEG | NEG | Concordance | Concordance | Concordance |
| A65 | Casablanca^1^ | 21 | NEG | NEG | NEG | Concordance | Concordance | Concordance |
| A66 | Casablanca^1^ | 19 | NEG | NEG | NEG | Concordance | Concordance | Concordance |
| A72 | Casablanca^1^ | 21 | NEG | NEG | NEG | Concordance | Concordance | Concordance |
| A76 | Casablanca^1^ | 18 | NEG | NEG | NEG | Concordance | Concordance | Concordance |
| A78 | Casablanca^1^ | 20 | NEG | NEG | NEG | Concordance | Concordance | Concordance |
| A82 | Casablanca^1^ |  | POS | POS | POS | Concordance | Concordance | Concordance |
| A84 | Casablanca^1^ | 20 | NEG | NEG | NEG | Concordance | Concordance | Concordance |
| 1 | Casablanca^2^ | 21 | NEG | NEG | NEG | Concordance | Concordance | Concordance |
| 2 | Casablanca^2^ | 25 | NEG | NEG | NEG | Concordance | Concordance | Concordance |
| 3 | Casablanca^2^ | 20 | POS | POS | POS | Concordance | Concordance | Concordance |
| 4 | Casablanca^2^ | 21 | NEG | NEG | NEG | Concordance | Concordance | Concordance |
| 5 | Casablanca^2^ | 26 | POS | POS | POS | Concordance | Concordance | Concordance |
| 6 | Casablanca^2^ | 19 | NEG | NEG | NEG | Concordance | Concordance | Concordance |
| 7 | Casablanca^2^ | 23 | NEG | NEG | NEG | Concordance | Concordance | Concordance |
| 8 | Casablanca^2^ | 22 | NEG | NEG | NEG | Concordance | Concordance | Concordance |
| 9 | Casablanca^2^ | 22 | NEG | NEG | NEG | Concordance | Concordance | Concordance |
| 10 | Casablanca^2^ | 20 | NEG | NEG | NEG | Concordance | Concordance | Concordance |
| 11 | Casablanca^2^ | 20 | NEG | NEG | NEG | Concordance | Concordance | Concordance |
| 12 | Casablanca^2^ | 20 | NEG | NEG | NEG | Concordance | Concordance | Concordance |
| 13 | Casablanca^2^ | 20 | NEG | NEG | NEG | Concordance | Concordance | Concordance |
| 15 | Casablanca^2^ | 20 | NEG | NEG | NEG | Concordance | Concordance | Concordance |
| 16 | Casablanca^2^ | 21 | NEG | NEG | NEG | Concordance | Concordance | Concordance |
| 17 | Casablanca^2^ | 21 | NEG | NEG | NEG | Concordance | Concordance | Concordance |
| 18 | Casablanca^2^ | 21 | NEG | NEG | NEG | Concordance | Concordance | Concordance |
| 19 | Casablanca^2^ | 20 | NEG | NEG | NEG | Concordance | Concordance | Concordance |
| 20 | Casablanca^2^ | 21 | NEG | NEG | NEG | Concordance | Concordance | Concordance |
| 21 | Casablanca^2^ | 20 | NEG | NEG | NEG | Concordance | Concordance | Concordance |
| A31 | Casablanca^2^ | 19 | NEG | NEG | NEG | Concordance | Concordance | Concordance |
| 24 | Casablanca^2^ | 21 | NEG | NEG | NEG | Concordance | Concordance | Concordance |
| 25 | Casablanca^2^ | 21 | NEG | NEG | NEG | Concordance | Concordance | Concordance |
| 26 | Casablanca^2^ | 22 | NEG | NEG | NEG | Concordance | Concordance | Concordance |
| 28 | Casablanca^2^ | 21 | NEG | NEG | NEG | Concordance | Concordance | Concordance |
| 29 | Casablanca^2^ | 21 | NEG | NEG | NEG | Concordance | Concordance | Concordance |
| 30 | Casablanca^2^ | 20 | NEG | NEG | NEG | Concordance | Concordance | Concordance |
| 31 | Casablanca^2^ | 21 | NEG | NEG | NEG | Concordance | Concordance | Concordance |
| 34 | Casablanca^2^ | 20 | NEG | NEG | NEG | Concordance | Concordance | Concordance |
| 35 | Casablanca^2^ | 42 | POS | POS | POS | Concordance | Concordance | Concordance |
| 37 | Casablanca^2^ | 18 | NEG | NEG | NEG | Concordance | Concordance | Concordance |
| 38 | Casablanca^2^ | 25 | NEG | NEG | NEG | Concordance | Concordance | Concordance |
| 39 | Casablanca^2^ | 35 | NEG | NEG | NEG | Concordance | Concordance | Concordance |
| 40 | Casablanca^2^ | 20 | NEG | NEG | NEG | Concordance | Concordance | Concordance |
| 41 | Casablanca^2^ | 54 | POS | POS | POS | Concordance | Concordance | Concordance |
| 42 | Casablanca^2^ | 18 | NEG | NEG | NEG | Concordance | Concordance | Concordance |
| 43 | Casablanca^2^ | 18 | NEG | NEG | NEG | Concordance | Concordance | Concordance |
| 44 | Casablanca^2^ | 18 | POS | POS | POS | Concordance | Concordance | Concordance |
| 45 | Casablanca^2^ | 17 | POS | POS | POS | Concordance | Concordance | Concordance |
| 46 | Casablanca^2^ | 18 | NEG | NEG | NEG | Concordance | Concordance | Concordance |
| 47 | Casablanca^2^ | 18 | NEG | NEG | NEG | Concordance | Concordance | Concordance |
| 48 | Casablanca^2^ | 19 | NEG | NEG | NEG | Concordance | Concordance | Concordance |
| 49 | Casablanca^2^ | 19 | POS | POS | POS | Concordance | Concordance | Concordance |
| 50 | Casablanca^2^ | 19 | NEG | NEG | NEG | Concordance | Concordance | Concordance |
| 51 | Casablanca^2^ | 31 | NEG | NEG | NEG | Concordance | Concordance | Concordance |
| 52 | Casablanca^2^ | 23 | POS | POS | POS | Concordance | Concordance | Concordance |
| 53 | Casablanca^2^ | 22 | NEG | NEG | NEG | Concordance | Concordance | Concordance |
| 54 | Casablanca^2^ | 21 | NEG | NEG | NEG | Concordance | Concordance | Concordance |
| 55 | Casablanca^2^ | 22 | NEG | NEG | NEG | Concordance | Concordance | Concordance |
| 56 | Casablanca^2^ | 20 | NEG | NEG | NEG | Concordance | Concordance | Concordance |
| 57 | Casablanca^2^ | 21 | NEG | NEG | NEG | Concordance | Concordance | Concordance |
| 58 | Casablanca^2^ | 18 | NEG | NEG | NEG | Concordance | Concordance | Concordance |
| 59 | Casablanca^2^ | 21 | NEG | NEG | NEG | Concordance | Concordance | Concordance |
| 60 | Casablanca^2^ | 21 | NEG | NEG | NEG | Concordance | Concordance | Concordance |
| 61 | Casablanca^2^ | 18 | NEG | NEG | NEG | Concordance | Concordance | Concordance |
| 62 | Casablanca^2^ | 38 | NEG | NEG | NEG | Concordance | Concordance | Concordance |
| 63 | Casablanca^2^ | 18 | POS | POS | POS | Concordance | Concordance | Concordance |
| 64 | Casablanca^2^ | 23 | NEG | NEG | NEG | Concordance | Concordance | Concordance |
| 65 | Casablanca^2^ | 19 | NEG | NEG | NEG | Concordance | Concordance | Concordance |
| 67 | Casablanca^2^ | 18 | NEG | NEG | NEG | Concordance | Concordance | Concordance |
| 70 | Casablanca^2^ | 18 | NEG | NEG | NEG | Concordance | Concordance | Concordance |
| 71 | Casablanca^2^ | 18 | NEG | NEG | NEG | Concordance | Concordance | Concordance |
| 72 | Casablanca^2^ | 21 | POS | POS | POS | Concordance | Concordance | Concordance |
| 73 | Casablanca^2^ | 21 | NEG | NEG | NEG | Concordance | Concordance | Concordance |
| 74 | Casablanca^2^ | 21 | NEG | NEG | NEG | Concordance | Concordance | Concordance |
| 75 | Casablanca^2^ | 19 | NEG | NEG | NEG | Concordance | Concordance | Concordance |
| 76 | Casablanca^2^ | 19 | NEG | NEG | NEG | Concordance | Concordance | Concordance |
| 77 | Casablanca^2^ | 19 | POS | POS | POS | Concordance | Concordance | Concordance |
| 78 | Casablanca^2^ | 19 | NEG | NEG | NEG | Concordance | Concordance | Concordance |
| 80 | Casablanca^2^ | 21 | POS | POS | POS | Concordance | Concordance | Concordance |
| 82 | Casablanca^2^ | 18 | NEG | NEG | NEG | Concordance | Concordance | Concordance |
| 83 | Casablanca^2^ | 25 | NEG | NEG | NEG | Concordance | Concordance | Concordance |
| 85 | Casablanca^2^ | 23 | NEG | NEG | NEG | Concordance | Concordance | Concordance |
| 86 | Casablanca^2^ | 20 | NEG | NEG | NEG | Concordance | Concordance | Concordance |
| 87 | Casablanca^2^ | 19 | NEG | NEG | NEG | Concordance | Concordance | Concordance |
| 88 | Casablanca^2^ | 22 | NEG | NEG | NEG | Concordance | Concordance | Concordance |
| 89 | Casablanca^2^ | 25 | POS | POS | POS | Concordance | Concordance | Concordance |
| 90 | Casablanca^2^ | 21 | NEG | NEG | NEG | Concordance | Concordance | Concordance |
| 91 | Casablanca^2^ | 21 | NEG | NEG | NEG | Concordance | Concordance | Concordance |
| 92 | Casablanca^2^ | 21 | NEG | NEG | NEG | Concordance | Concordance | Concordance |
| 93 | Casablanca^2^ | 20 | NEG | NEG | NEG | Concordance | Concordance | Concordance |
| 94 | Casablanca^2^ | 20 | NEG | NEG | NEG | Concordance | Concordance | Concordance |
| 95 | Casablanca^2^ | 21 | POS | POS | POS | Concordance | Concordance | Concordance |
| 98 | Casablanca^2^ | 27 | NEG | NEG | NEG | Concordance | Concordance | Concordance |
| 99 | Casablanca^2^ | 33 | NEG | NEG | NEG | Concordance | Concordance | Concordance |
| 117 | Casablanca^2^ | 42 | NEG | NEG | NEG | Concordance | Concordance | Concordance |
| 101 | Casablanca^2^ | 23 | NEG | NEG | NEG | Concordance | Concordance | Concordance |
| 102 | Casablanca^2^ | 18 | NEG | NEG | NEG | Concordance | Concordance | Concordance |
| 103 | Casablanca^2^ | 18 | NEG | NEG | NEG | Concordance | Concordance | Concordance |
| A32 | Casablanca^2^ | 18 | NEG | NEG | NEG | Concordance | Concordance | Concordance |
| 105 | Casablanca^2^ | 40 | NEG | NEG | NEG | Concordance | Concordance | Concordance |
| 106 | Casablanca^2^ | 20 | POS | POS | POS | Concordance | Concordance | Concordance |
| 107 | Casablanca^2^ | 19 | NEG | NEG | NEG | Concordance | Concordance | Concordance |
| 108 | Casablanca^2^ | 20 | NEG | NEG | NEG | Concordance | Concordance | Concordance |
| 109 | Casablanca^2^ | 25 | POS | POS | POS | Concordance | Concordance | Concordance |
| 110 | Casablanca^2^ | 20 | POS | POS | POS | Concordance | Concordance | Concordance |
| 111 | Casablanca^2^ | 22 | NEG | NEG | NEG | Concordance | Concordance | Concordance |
| 112 | Casablanca^2^ | 18 | NEG | NEG | NEG | Concordance | Concordance | Concordance |
| A39 | Casablanca^2^ | 18 | NEG | NEG | NEG | Concordance | Concordance | Concordance |
| 114 | Casablanca^2^ | 22 | NEG | NEG | NEG | Concordance | Concordance | Concordance |
| 115 | Casablanca^2^ | 20 | NEG | NEG | NEG | Concordance | Concordance | Concordance |
| 116 | Casablanca^2^ | 24 | NEG | NEG | NEG | Concordance | Concordance | Concordance |
| 117 | Casablanca^2^ | 42 | NEG | NEG | NEG | Concordance | Concordance | Concordance |
| T57 | Rabat^3^ | 48 | POS | POS | POS | Concordance | Concordance | Concordance |
| T58 | Rabat^3^ | 27 | NEG | NEG | NEG | Concordance | Concordance | Concordance |
| T59 | Rabat^3^ | 33 | NEG | NEG | NEG | Concordance | Concordance | Concordance |
| T21 | Rabat^3^ |  | NEG | NEG | NEG | Concordance | Concordance | Concordance |
| T61 | Rabat^3^ | 30 | NEG | NEG | NEG | Concordance | Concordance | Concordance |
| T62 | Rabat^3^ | 40 | NEG | NEG | NEG | Concordance | Concordance | Concordance |
| T63 | Rabat^3^ | 54 | POS | POS | POS | Concordance | Concordance | Concordance |
| T64 | Rabat^3^ | 45 | POS | POS | POS | Concordance | Concordance | Concordance |
| T23 | Rabat^3^ |  | NEG | NEG | NEG | Concordance | Concordance | Concordance |
| T66 | Rabat^3^ | 70 | NEG | NEG | NEG | Concordance | Concordance | Concordance |
| T67 | Rabat^3^ | 43 | NEG | NEG | NEG | Concordance | Concordance | Concordance |
| T68 | Rabat^3^ | 27 | NEG | NEG | NEG | Concordance | Concordance | Concordance |
| T69 | Rabat^3^ | 40 | POS | POS | POS | Concordance | Concordance | Concordance |
| T70 | Rabat^3^ | 40 | POS | POS | POS | Concordance | Concordance | Concordance |
| T24 | Rabat^3^ |  | POS | POS | POS | Concordance | Concordance | Concordance |
| T72 | Rabat^3^ | 60 | NEG | POS | POS | **Disconcordance** | **Disconcordance** | Concordance |
| T73 | Rabat^3^ | 54 | POS | POS | POS | Concordance | Concordance | Concordance |
| T74 | Rabat^3^ | 64 | POS | POS | POS | Concordance | Concordance | Concordance |
| T52 | Rabat^3^ | 34 | POS | POS | POS | Concordance | Concordance | Concordance |
| T76 | Rabat^3^ | 27 | NEG | NEG | NEG | Concordance | Concordance | Concordance |
| T77 | Rabat^3^ | 34 | NEG | NEG | NEG | Concordance | Concordance | Concordance |
| T78 | Rabat^3^ | 31 | POS | POS | POS | Concordance | Concordance | Concordance |
| T79 | Rabat^3^ | 67 | POS | POS | POS | Concordance | Concordance | Concordance |
| T80 | Rabat^3^ | 42 | POS | POS | POS | Concordance | Concordance | Concordance |
| T27 | Rabat^3^ | 48 | POS | POS | POS | Concordance | Concordance | Concordance |
| T82 | Rabat^3^ | 38 | POS | POS | POS | Concordance | Concordance | Concordance |
| T83 | Rabat^3^ | 40 | POS | POS | POS | Concordance | Concordance | Concordance |
| T84 | Rabat^3^ | 66 | NEG | NEG | NEG | Concordance | Concordance | Concordance |
| T85 | Rabat^3^ | 25 | NEG | NEG | NEG | Concordance | Concordance | Concordance |
| T86 | Rabat^3^ | 47 | POS | POS | POS | Concordance | Concordance | Concordance |
| T87 | Rabat^3^ | 34 | POS | POS | POS | Concordance | Concordance | Concordance |
| T88 | Rabat^3^ | 24 | POS | POS | POS | Concordance | Concordance | Concordance |
| T89 | Rabat^3^ | 58 | POS | POS | POS | Concordance | Concordance | Concordance |
| T90 | Rabat^3^ | 70 | NEG | NEG | NEG | Concordance | Concordance | Concordance |
| T91 | Rabat^3^ | 63 | POS | POS | POS | Concordance | Concordance | Concordance |
| T92 | Rabat^3^ | 30 | POS | POS | POS | Concordance | Concordance | Concordance |
| T93 | Rabat^3^ | 70 | POS | POS | POS | Concordance | Concordance | Concordance |
| T94 | Rabat^3^ | 48 | POS | POS | POS | Concordance | Concordance | Concordance |
| T35 | Rabat^3^ | 55 | NEG | POS | POS | **Disconcordance** | **Disconcordance** | Concordance |
| T96 | Rabat^3^ | 46 | POS | POS | POS | Concordance | Concordance | Concordance |
| T97 | Rabat^3^ | 21 | POS | POS | POS | Concordance | Concordance | Concordance |
| T98 | Rabat^3^ | 40 | NEG | NEG | NEG | Concordance | Concordance | Concordance |
| T99 | Rabat^3^ | 47 | NEG | NEG | NEG | Concordance | Concordance | Concordance |
| T100 | Rabat^3^ | 16 | NEG | NEG | NEG | Concordance | Concordance | Concordance |
| T101 | Rabat^3^ | 40 | POS | POS | POS | Concordance | Concordance | Concordance |
| T102 | Rabat^3^ | 60 | POS | POS | POS | Concordance | Concordance | Concordance |
| T103 | Rabat^3^ | 35 | NEG | NEG | NEG | Concordance | Concordance | Concordance |
| T104 | Rabat^3^ | 22 | NEG | NEG | NEG | Concordance | Concordance | Concordance |
| T105 | Rabat^3^ | 50 | POS | POS | POS | Concordance | Concordance | Concordance |
| T53 | Rabat^3^ | 29 | POS | POS | POS | Concordance | Concordance | Concordance |
| T54 | Rabat^3^ | 30 | NEG | NEG | NEG | Concordance | Concordance | Concordance |
| T108 | Rabat^3^ | 50 | POS | POS | POS | Concordance | Concordance | Concordance |
| T109 | Rabat^3^ | 50 | POS | POS | POS | Concordance | Concordance | Concordance |
| T110 | Rabat^3^ | 23 | POS | POS | POS | Concordance | Concordance | Concordance |
| T111 | Rabat^3^ | 43 | POS | POS | POS | Concordance | Concordance | Concordance |
| T112 | Rabat^3^ | 40 | POS | POS | POS | Concordance | Concordance | Concordance |
| T113 | Rabat^3^ | 41 | POS | POS | POS | Concordance | Concordance | Concordance |
| T114 | Rabat^3^ | 39 | POS | POS | POS | Concordance | Concordance | Concordance |
| T115 | Rabat^3^ | 59 | POS | POS | POS | Concordance | Concordance | Concordance |
| T116 | Rabat^3^ | 53 | POS | POS | POS | Concordance | Concordance | Concordance |
| T117 | Rabat^3^ | 58 | POS | POS | POS | Concordance | Concordance | Concordance |
| T118 | Rabat^3^ | 50 | NEG | NEG | NEG | Concordance | Concordance | Concordance |
| T119 | Rabat^3^ | 52 | POS | NEG | NEG | **Disconcordance** | **Disconcordance** | Concordance |
| T120 | Rabat^3^ | 41 | NEG | NEG | NEG | Concordance | Concordance | Concordance |
| T121 | Rabat^3^ | 34 | POS | POS | POS | Concordance | Concordance | Concordance |
| T122 | Rabat^3^ | 39 | NEG | NEG | NEG | Concordance | Concordance | Concordance |
| T123 | Rabat^3^ | 57 | POS | POS | POS | Concordance | Concordance | Concordance |
| T124 | Rabat^3^ | 20 | NEG | NEG | NEG | Concordance | Concordance | Concordance |
| T125 | Rabat^3^ | 38 | POS | POS | POS | Concordance | Concordance | Concordance |
| T126 | Rabat^3^ | 54 | POS | POS | POS | Concordance | Concordance | Concordance |
| T127 | Rabat^3^ | 57 | POS | POS | POS | Concordance | Concordance | Concordance |
| T128 | Rabat^3^ | 22 | NEG | NEG | NEG | Concordance | Concordance | Concordance |
| T129 | Rabat^3^ | 25 | POS | POS | POS | Concordance | Concordance | Concordance |
| T130 | Rabat^3^ | 26 | NEG | NEG | NEG | Concordance | Concordance | Concordance |
| T18 | Rabat^3^ | 23 | NEG | NEG | NEG | Concordance | Concordance | Concordance |
| T132 | Rabat^3^ | 18 | NEG | NEG | NEG | Concordance | Concordance | Concordance |
| T133 | Rabat^3^ | 24 | NEG | NEG | NEG | Concordance | Concordance | Concordance |
| T16 | Rabat^3^ | 21 | POS | POS | POS | Concordance | Concordance | Concordance |
| T135 | Rabat^3^ | 21 | NEG | NEG | NEG | Concordance | Concordance | Concordance |
| T136 | Rabat^3^ | 30 | NEG | NEG | NEG | Concordance | Concordance | Concordance |
| T137 | Rabat^3^ | 50 | POS | POS | POS | Concordance | Concordance | Concordance |
| T138 | Rabat^3^ | 28 | NEG | NEG | NEG | Concordance | Concordance | Concordance |
| T139 | Rabat^3^ | 31 | NEG | NEG | NEG | Concordance | Concordance | Concordance |
| T140 | Rabat^3^ | 30 | POS | POS | POS | Concordance | Concordance | Concordance |
| T141 | Rabat^3^ | 34 | NEG | NEG | NEG | Concordance | Concordance | Concordance |
| T142 | Rabat^3^ | 46 | NEG | NEG | NEG | Concordance | Concordance | Concordance |
| T143 | Rabat^3^ | 41 | NEG | NEG | NEG | Concordance | Concordance | Concordance |
| T144 | Rabat^3^ | 44 | NEG | NEG | NEG | Concordance | Concordance | Concordance |
| T145 | Rabat^3^ | 56 | POS | POS | POS | Concordance | Concordance | Concordance |
| T146 | Rabat^3^ | 43 | NEG | NEG | NEG | Concordance | Concordance | Concordance |
| T147 | Rabat^3^ | 24 | NEG | NEG | NEG | Concordance | Concordance | Concordance |
| T148 | Rabat^3^ | 22 | NEG | NEG | NEG | Concordance | Concordance | Concordance |
| T149 | Rabat^3^ | 52 | NEG | NEG | NEG | Concordance | Concordance | Concordance |
| T150 | Rabat^3^ | 55 | POS | POS | POS | Concordance | Concordance | Concordance |
| T151 | Rabat^3^ | 60 | POS | POS | POS | Concordance | Concordance | Concordance |
| T152 | Rabat^3^ | 20 | NEG | NEG | NEG | Concordance | Concordance | Concordance |
| T17 | Rabat^3^ |  | NEG | NEG | NEG | Concordance | Concordance | Concordance |
| T154 | Rabat^3^ | 44 | NEG | NEG | NEG | Concordance | Concordance | Concordance |
| T19 | Rabat^3^ | 32 | POS | POS | POS | Concordance | Concordance | Concordance |
| T156 | Rabat^3^ | 62 | POS | POS | POS | Concordance | Concordance | Concordance |
| T157 | Rabat^3^ | 60 | POS | POS | POS | Concordance | Concordance | Concordance |
| T158 | Rabat^3^ | 40 | NEG | NEG | NEG | Concordance | Concordance | Concordance |
| T159 | Rabat^3^ | 38 | POS | POS | POS | Concordance | Concordance | Concordance |
| T160 | Rabat^3^ | 24 | POS | POS | POS | Concordance | Concordance | Concordance |
| T161 | Rabat^3^ | 49 | NEG | NEG | NEG | Concordance | Concordance | Concordance |
| T162 | Rabat^3^ | 30 | NEG | NEG | NEG | Concordance | Concordance | Concordance |
| T163 | Rabat^3^ | 59 | POS | POS | POS | Concordance | Concordance | Concordance |
| T164 | Rabat^3^ | 34 | POS | POS | POS | Concordance | Concordance | Concordance |
| T165 | Rabat^3^ | 60 | POS | POS | POS | Concordance | Concordance | Concordance |
| T15 | Rabat^3^ | 56 | NEG | POS | POS | **Disconcordance** | **Disconcordance** | Concordance |
| T167 | Rabat^3^ | 37 | NEG | NEG | NEG | Concordance | Concordance | Concordance |
| T168 | Rabat^3^ | 57 | POS | POS | POS | Concordance | Concordance | Concordance |
| T169 | Rabat^3^ | 52 | NEG | NEG | NEG | Concordance | Concordance | Concordance |
| T170 | Rabat^3^ | 51 | POS | POS | POS | Concordance | Concordance | Concordance |
| T171 | Rabat^3^ | 35 | POS | POS | POS | Concordance | Concordance | Concordance |
| T172 | Rabat^3^ | 26 | NEG | NEG | NEG | Concordance | Concordance | Concordance |
| T20 | Rabat^3^ | 29 | POS | POS | POS | Concordance | Concordance | Concordance |
| T174 | Rabat^3^ | 25 | NEG | NEG | NEG | Concordance | Concordance | Concordance |
| T11 | Rabat^3^ | 57 | POS | POS | POS | Concordance | Concordance | Concordance |
| T176 | Rabat^3^ | 40 | NEG | NEG | NEG | Concordance | Concordance | Concordance |
| T07 | Rabat^3^ | 55 | POS | POS | POS | Concordance | Concordance | Concordance |
| T178 | Rabat^3^ | 54 | POS | POS | POS | Concordance | Concordance | Concordance |
| T179 | Rabat^3^ | 45 | NEG | NEG | NEG | Concordance | Concordance | Concordance |
| T08 | Rabat^3^ | 39 | NEG | NEG | NEG | Concordance | Concordance | Concordance |
| T181 | Rabat^3^ | 46 | POS | POS | POS | Concordance | Concordance | Concordance |
| T182 | Rabat^3^ | 20 | NEG | NEG | NEG | Concordance | Concordance | Concordance |
| T183 | Rabat^3^ | 21 | POS | POS | POS | Concordance | Concordance | Concordance |
| T184 | Rabat^3^ | 30 | POS | POS | POS | Concordance | Concordance | Concordance |
| T185 | Rabat^3^ | 44 | POS | POS | POS | Concordance | Concordance | Concordance |
| T186 | Rabat^3^ | 24 | NEG | NEG | NEG | Concordance | Concordance | Concordance |
| T187 | Rabat^3^ | 20 | POS | POS | POS | Concordance | Concordance | Concordance |
| T188 | Rabat^3^ | 20 | POS | POS | POS | Concordance | Concordance | Concordance |
| T189 | Rabat^3^ | 35 | POS | POS | POS | Concordance | Concordance | Concordance |
| T04 | Rabat^3^ | 55 | NEG | POS | POS | **Disconcordance** | **Disconcordance** | Concordance |
| T12 | Rabat^3^ | 28 | POS | POS | POS | Concordance | Concordance | Concordance |
| T192 | Rabat^3^ | 23 | POS | POS | POS | Concordance | Concordance | Concordance |
