# Supplementary material for: High performance of a novel point-of-care blood test for Toxoplasma infection in women from diverse regions of Morocco
Source: Emerg Microbes Infect. 2021 Aug 22;10(1):1675–82. doi: 10.1080/22221751.2021.1948359 (PMC8381951; doi:10.1080/22221751.2021.1948359)
Supplement: Table_1S.docx [file TEMI_A_1948359_SM9340.docx]

**Supporting Information**

**Supplemental Table S1**

| **Province** | **Participant Number** | **Age** | **Result of POC Test** | **IgG** | **IgM** | **Pregnant** | **Others** |
| --- | --- | --- | --- | --- | --- | --- | --- |
| Rabat | 1 | 39 | POS | POS | NEG | NO |  |
| Rabat | T4 | 55 | NEG | NEG | NEG | NO |  |
| Rabat | T5 | 36 | POS | POS | NEG | NO |  |
| Rabat | T6 | 36 | POS | POS | NEG | NO |  |
| Rabat | T7 | 55 | POS | POS | NEG | NO |  |
| Rabat | T8 | 39 | NEG | NEG | NEG | NO |  |
| Rabat | T9 | 52 | POS | POS | NEG | NO |  |
| Rabat | T10 | 33 | POS | POS | NEG | NO |  |
| Rabat | T11 | 57 | POS | POS | NEG | NO |  |
| Rabat | T12 | 28 | POS | POS | NEG | NO |  |
| Rabat | T13 | 21 | NEG | NEG | NEG | YES |  |
| Rabat | T14 | 36 | POS | POS | NEG | ND |  |
| Rabat | T15 | 56 | NEG | NEG | POS | NO |  |
| Rabat | T16 | 21 | POS | POS | POS | NO |  |
| Rabat | T19 | 32 | POS | POS | NEG | ND |  |
| Rabat | T20 | 29 | POS | POS | NEG | NO |  |
| Rabat | T21 | 35 | NEG | NEG | NEG | NO |  |
| Rabat | T25 | 29 | POS | POS | POS | NO |  |
| Rabat | T26 | 45 | NEG | NEG | NEG | NO |  |
| Rabat | T27 | 48 | POS | POS | NEG | NO |  |
| Rabat | T28 | 55 | NEG | NEG | NEG | ND |  |
| Rabat | T29 | ND | POS | POS | NEG | NO |  |
| Rabat | T30 | 32 | NEG | NEG | NEG | NO |  |
| Rabat | T31 | 34 | POS | POS | POS | YES |  |
| Rabat | T32 | 38 | POS | POS | NEG | YES |  |
| Rabat | T33 | 61 | POS | POS | NEG | NO |  |
| Rabat | T34 | 42 | POS | POS | NEG | NO |  |
| Rabat | T35 | 55 | NEG | POS | NEG | NO |  |
| Rabat | T36 | 35 | NEG | NEG | NEG | NO |  |
| Rabat | T37 | 58 | POS | POS | ND | NO |  |
| Rabat | T38 | 56 | NEG | NEG | NEG | NO |  |
| Rabat | T39 | 58 | NEG | NEG | NEG | NO |  |
| Rabat | T40 | 30 | NEG | NEG | NEG | YES |  |
| Rabat | T41 | 21 | POS | POS | NEG | NO |  |
| Rabat | T42 | 41 | POS | POS | NEG | NO |  |
| Rabat | T43 | 42 | POS | POS | NEG | NO |  |
| Rabat | T44 | 37 | NEG | NEG | NEG | NO |  |
| Rabat | T45 | 17 | NEG | NEG | NEG | YES |  |
| Rabat | T46 | 57 | POS | POS | NEG | NO |  |
| Rabat | T47 | 29 | POS | POS | POS | NO |  |
| Rabat | T48 | 15 | POS | POS | NEG | NO |  |
| Rabat | T49 | 46 | NEG | NEG | NEG | NO |  |
| Rabat | T50 | 26 | NEG | NEG | NEG | YES | ~6 months pregnant |
| Rabat | T51 | 25 | NEG | NEG | NEG | NO |  |
| Rabat | T52 | 34 | POS | POS | NEG | YES | ND |
| Rabat | T53 | 29 | POS | POS | NEG | YES | ND |
| Rabat | T54 | 30 | NEG | NEG | NEG | NO |  |
| Rabat | T55 | 28 | NEG | NEG | NEG | NO |  |
| Rabat | T56 | 27 | NEG | NEG | NEG | NO |  |
| Rabat | T57 | 48 | POS | POS | NEG | NO |  |
| Rabat | T58 | 27 | NEG | NEG | NEG | NO |  |
| Rabat | T59 | 33 | NEG | NEG | NEG | NO |  |
| Rabat | T60 | 34 | POS | POS | NEG | NO |  |
| Rabat | T61 | 30 | NEG | NEG | NEG | NO |  |
| Rabat | T62 | 40 | NEG | NEG | NEG | NO |  |
| Rabat | T63 | 54 | POS | POS | NEG | NO |  |
| Rabat | T64 | 45 | POS | POS | ND | NO |  |
| Rabat | T65 | 17 | POS | POS | ND | NO |  |
| Rabat | T66 | 70 | NEG | NEG | NEG | NO |  |
| Rabat | T67 | 43 | NEG | NEG | ND | NO |  |
| Rabat | T68 | 27 | NEG | NEG | NEG | YES | ~4 months pregnant |
| Rabat | T69 | 40 | POS | POS | NEG | YES | ~5 months pregnant |
| Rabat | T70 | 40 | POS | POS | ND | NO |  |
| Rabat | T71 | 29 | NEG | NEG | NEG | NO |  |
| Rabat | T72 | 60 | NEG | NEG | NEG | NO |  |
| Rabat | T73 | 54 | POS | POS | NEG | NO |  |
| Rabat | T74 | 64 | POS | POS | NEG | NO |  |
| Rabat | T75 | - | NEG | NEG | NEG | NO |  |
| Rabat | T76 | 27 | NEG | NEG | ND | NO |  |
| Rabat | T77 | 34 | NEG | NEG | NEG | NO |  |
| Rabat | T78 | 31 | POS | POS | NEG | NO |  |
| Rabat | T79 | 67 | POS | POS | ND | NO |  |
| Rabat | T80 | 42 | POS | POS | NEG | NO |  |
| Rabat | T81 | 44 | NEG | NEG | NEG | NO |  |
| Rabat | T82 | 38 | POS | POS | ND | ND |  |
| Rabat | T83 | 40 | POS | POS | ND | YES | ~6 months pregnant |
| Rabat | T84 | 66 | NEG | NEG | NEG | NO |  |
| Rabat | T85 | 25 | NEG | NEG | NEG | NO |  |
| Rabat | T86 | 47 | POS | POS | POS | NO |  |
| Rabat | T87 | 34 | POS | POS | ND | NO |  |
| Rabat | T88 | 24 | POS | POS | NEG | NO |  |
| Rabat | T89 | 58 | POS | POS | NEG | NO |  |
| Rabat | T90 | 70 | NEG | NEG | NEG | NO |  |
| Rabat | T91 | 63 | POS | POS | NEG | NO |  |
| Rabat | T92 | 30 | POS | POS | NEG | NO |  |
| Rabat | T93 | 70 | POS | POS | NEG | NO |  |
| Rabat | T94 | 48 | POS | POS | NEG | NO |  |
| Rabat | T95 | 33 | POS | POS | NEG | NO |  |
| Rabat | T96 | 46 | POS | POS | NEG | NO |  |
| Rabat | T97 | 21 | POS | POS | NEG | NO |  |
| Rabat | T98 | 40 | NEG | NEG | NEG | NO |  |
| Rabat | T99 | 47 | NEG | NEG | NEG | NO |  |
| Rabat | T100 | 16 | NEG | NEG | NEG | NO |  |
| Rabat | T101 | 40 | POS | POS | ND | NO |  |
| Rabat | T102 | 60 | POS | POS | NEG | NO |  |
| Rabat | T103 | 35 | NEG | NEG | NEG | NO |  |
| Rabat | T104 | 22 | NEG | NEG | NEG | YES | ~4 months pregnant |
| Rabat | T105 | 50 | POS | POS | NEG | NO |  |
| Rabat | T106 | 20 | POS | POS | ND | NO |  |
| Rabat | T107 | 20 | NEG | NEG | ND | NO |  |
| Rabat | T108 | 50 | POS | POS | NEG | NO |  |
| Rabat | T109 | 50 | POS | POS | ND | NO |  |
| Rabat | T110 | 23 | POS | POS | ND | YES | >3 months pregnant |
| Rabat | T111 | 43 | POS | POS | ND | NO |  |
| Rabat | T112 | 40 | POS | POS | ND | NO |  |
| Rabat | T113 | 41 | POS | POS | ND | NO |  |
| Rabat | T114 | 39 | POS | POS | ND | NO |  |
| Rabat | T115 | 59 | POS | POS | ND | NO |  |
| Rabat | T116 | 53 | POS | POS | NEG | NO |  |
| Rabat | T117 | 58 | POS | POS | NEG | NO |  |
| Rabat | T118 | 50 | NEG | NEG | ND | NO |  |
| Rabat | T119 | 52 | POS | POS | ND | - |  |
| Rabat | T120 | 41 | NEG | NEG | ND | NO |  |
| Rabat | T121 | 34 | POS | POS | ND | - |  |
| Rabat | T122 | 39 | NEG | NEG | ND | NO |  |
| Rabat | T123 | 57 | POS | POS | ND | NO |  |
| Rabat | T124 | 20 | NEG | NEG | ND | NO |  |
| Rabat | T125 | 38 | POS | POS | ND | NO |  |
| Rabat | T126 | 54 | POS | POS | ND | NO |  |
| Rabat | T127 | 57 | POS | POS | ND | NO |  |
| Rabat | T128 | 22 | NEG | NEG | NEG | YES | ~5 months pregnant |
| Rabat | T129 | 25 | POS | POS | NEG | NO |  |
| Rabat | T130 | 26 | NEG | NEG | NEG | NO |  |
| Rabat | T131 | 42 | POS | POS | POS | NO |  |
| Rabat | T132 | 18 | NEG | NEG | ND | NO |  |
| Rabat | T133 | 24 | NEG | NEG | NEG | NO |  |
| Rabat | T134 | 52 | POS | POS | NEG | NO |  |
| Rabat | T135 | 21 | NEG | NEG | NEG | NO |  |
| Rabat | T136 | 30 | NEG | NEG | NEG | NO |  |
| Rabat | T137 | 50 | POS | POS | NEG | NO |  |
| Rabat | T138 | 28 | NEG | NEG | NEG | NO |  |
| Rabat | T139 | 31 | NEG | NEG | NEG | NO |  |
| Rabat | T140 | 30 | POS | POS | NEG | NO |  |
| Rabat | T141 | 34 | NEG | NEG | NEG | YES | ~3 months pregnant |
| Rabat | T142 | 46 | NEG | NEG | NEG | NO |  |
| Rabat | T143 | 41 | NEG | NEG | NEG | YES | ~3 months pregnant |
| Rabat | T144 | 44 | NEG | NEG | NEG | NO |  |
| Rabat | T145 | 56 | POS | POS | NEG | NO |  |
| Rabat | T146 | 43 | NEG | NEG | NEG | NO |  |
| Rabat | T147 | 24 | NEG | NEG | NEG | NO |  |
| Rabat | T148 | 22 | NEG | NEG | NEG | YES | ND |
| Rabat | T149 | 52 | NEG | NEG | NEG | NO |  |
| Rabat | T150 | 55 | POS | POS | NEG | NO |  |
| Rabat | T151 | 60 | POS | POS | NEG | NO |  |
| Rabat | T152 | 20 | NEG | NEG | NEG | NO |  |
| Rabat | T153 | 44 | NEG | NEG | NEG | NO |  |
| Rabat | T154 | 44 | NEG | NEG | NEG | NO |  |
| Rabat | T155 | 50 | NEG | NEG | NEG | NO |  |
| Rabat | T156 | 62 | POS | POS | NEG | NO |  |
| Rabat | T157 | 60 | POS | POS | NEG | NO |  |
| Rabat | T158 | 40 | NEG | NEG | NEG | NO |  |
| Rabat | T159 | 38 | POS | POS | NEG | NO |  |
| Rabat | T160 | 24 | POS | POS | NEG | YES | ~3 months pregnant |
| Rabat | T161 | 49 | NEG | NEG | NEG | NO |  |
| Rabat | T162 | 30 | NEG | NEG | NEG | YES | >7 months pregnant |
| Rabat | T163 | 59 | POS | POS | NEG | NO |  |
| Rabat | T164 | 34 | POS | POS | NEG | NO |  |
| Rabat | T165 | 60 | POS | POS | NEG | NO |  |
| Rabat | T166 | 21 | NEG | NEG | ND | NO |  |
| Rabat | T167 | 37 | NEG | NEG | NEG | NO |  |
| Rabat | T168 | 57 | POS | POS | NEG | NO |  |
| Rabat | T169 | 52 | NEG | NEG | NEG | NO |  |
| Rabat | T170 | 51 | POS | POS | NEG | NO |  |
| Rabat | T171 | 35 | POS | POS | NEG | NO |  |
| Rabat | T172 | 26 | NEG | NEG | NEG | - |  |
| Rabat | T173 | 28 | NEG | NEG | ND | NO |  |
| Rabat | T174 | 25 | NEG | NEG | NEG | NO |  |
| Rabat | T175 | 19 | NEG | NEG | ND | NO |  |
| Rabat | T176 | 40 | NEG | NEG | NEG | NO |  |
| Rabat | T177 | 43 | NEG | NEG | ND | NO |  |
| Rabat | T178 | 54 | POS | POS | NEG | NO |  |
| Rabat | T179 | 45 | NEG | NEG | NEG | NO |  |
| Rabat | T180 | 46 | POS | POS | ND | NO |  |
| Rabat | T181 | 46 | POS | POS | NEG | NO |  |
| Rabat | T182 | 20 | NEG | NEG | NEG | NO |  |
| Rabat | T183 | 21 | POS | POS | NEG | YES | >8 months pregnant |
| Rabat | T184 | 30 | POS | POS | NEG | NO |  |
| Rabat | T185 | 44 | POS | POS | NEG | NO |  |
| Rabat | T186/19 | 24 | NEG | NEG | NEG | - | - |
| Rabat | T187 | 20 | POS | POS | NEG | - | - |
| Rabat | T189 | 35 | POS | POS | NEG | NO | - |
| Rabat | T190 | 36 | POS | POS | NEG | NO | - |
| Rabat | T191 | 48 | POS | POS | NEG | NO | - |
| Rabat | T192 | 23 | POS | POS | NEG | NO | - |
| Casablanca | C01 | 22 | POS | POS | NEG | YES | - |
| Casablanca | CO2 | 38 | POS | POS | NEG | NO | - |
| Casablanca | CO3 | 25 | POS | POS | NEG | NO | - |
| Casablanca | CO4 | 30 | POS | POS | NEG | YES | - |
| Casablanca | CO5 | 41 | NEG | NEG | NEG | NO | - |
| Casablanca | CO6 | 47 | POS | POS | NEG | NO | - |
| Casablanca | CO7 | 30 | POS | POS | NEG | NO | - |
| Casablanca | CO8 | 21 | POS | POS | NEG | YES | - |
| Casablanca | CO9 | 27 | NEG | NEG | NEG | YES | - |
| Casablanca | C10 | 49 | POS | POS | NEG | NO | - |
| Casablanca | C11 | 63 | POS | POS | NEG | NO | - |
| Casablanca | C12 | 64 | NEG | POS | NEG | NO | - |
| Casablanca | C13 | 25 | NEG | POS | NEG | YES | - |
| Casablanca | C14 | 61 | NEG | NEG | NEG | NO | - |
| Casablanca | C15 | 49 | POS | POS | NEG | NO | - |
| Casablanca | C16 | 54 | NEG | NEG | NEG | NO | - |
| Casablanca | C17 | 38 | NEG | NEG | NEG | NO | - |
| Casablanca | C18 | 20 | POS | POS | NEG | YES | - |
| Casablanca | C19 | 36 | NEG | NEG | NEG | YES | - |
| Casablanca | C20 | 55 | NEG | POS | NEG | NO | - |
| Casablanca | C21 | 31 | NEG | NEG | NEG | YES | - |
| Casablanca | C22 | 30 | POS | POS | POS | YES | - |
| Casablanca | C23 | 32 | NEG | NEG | NEG | YES | - |
| Casablanca | C24 | 27 | NEG | NEG | NEG | YES | - |
| Casablanca | C25 | 27 | NEG | NEG | NEG | YES | - |
| Casablanca | C26 | 38 | POS | POS | NEG | NO | - |
| Casablanca | C27 | 21 | POS | POS | POS | YES | - |
| Casablanca | C28 | 24 | NEG | NEG | NEG | YES | - |
| Casablanca | C29 | 27 | POS | POS | NEG | NO | - |
| Casablanca | C30 | 20 | POS | POS | POS | NO | - |
| Casablanca | C31 | 23 | NEG | NEG | NEG | NO | - |
| Casablanca | C32 | 19 | NEG | NEG | NEG | NO | - |
| Casablanca | C33 | 21 | NEG | NEG | NEG | NO | - |
| Casablanca | C34 | 22 | NEG | NEG | NEG | NO | - |
| Casablanca | C35 | 20 | NEG | NEG | NEG | NO | - |
| Casablanca | C36 | 19 | NEG | NEG | ND | NO | - |
| Tinghir | H01/18 | 30 | POS | POS | NEG | YES | >8 months pregnant |
| Tinghir | H02/18 | 54 | NEG | NEG | NEG | NO |  |
| Tinghir | H03/18 | 28 | NEG | NEG | NEG | YES | ~7 months pregnant |
| Tinghir | H04/18 | 24 | POS | POS | NEG | YES | ~3 months pregnant |
| Tinghir | H05/18 | 34 | POS | POS | NEG | NO |  |
| Tinghir | H06/18 | 33 | NEG | NEG | NEG | YES | ND |
| Tinghir | H07/18 | 41 | NEG | NEG | NEG | NO |  |
| Tinghir | H08/18 | 30 | NEG | NEG | NEG | NO |  |
| Tinghir | H09/18 | 61 | POS | POS | NEG | NO |  |
| Tinghir | H10/18 | 25 | NEG | NEG | NEG | YES | ND |
| Tinghir | H11/18 | 60 | NEG | NEG | NEG | NO |  |
| Tinghir | H12/18 | 34 | NEG | NEG | NEG | YES | ND |
| Tinghir | H13/18 | 43 | NEG | NEG | NEG | YES | ND |
| Tinghir | H14/18 | 29 | NEG | NEG | NEG | NO |  |
| Tinghir | H15/18 | 27 | NEG | NEG | NEG | NO |  |
| Tinghir | H16/18 | 26 | NEG | NEG | NEG | NO |  |
| Tinghir | H17/18 | 54 | POS | POS | NEG | NO |  |
| Tinghir | H18/18 | 29 | POS | POS | NEG | NO |  |
| Tinghir | H19/18 | 33 | POS | POS | NEG | NO |  |
| Tinghir | H20/18 | 40 | POS | POS | NEG | YES | ND |
| Tinghir | H21/18 | 30 | POS | POS | NEG | YES | ND |
| Tinghir | H22/18 | 33 | NEG | NEG | NEG | YES | ND |
| Tinghir | H23/18 | 30 | NEG | NEG | NEG | YES | ND |
| Tinghir | H24/18 | 21 | NEG | NEG | NEG | NO |  |
| Tinghir | H25/18 | 37 | NEG | NEG | NEG | YES | ND |
| Tinghir | H26/18 | 23 | POS | POS | NEG | NO |  |
| Tinghir | H27/18 | 40 | POS | POS | NEG | YES | ND |
| Tinghir | H28/18 | - | NEG | NEG | NEG | NEG |  |
| Tinghir | H29/18 | 28 | POS | POS | POS | YES | ND |
| Tinghir | H30/18 | - | NEG | NEG | NEG | YES | ND |
| Tinghir | H31/18 | 30 | POS | POS | POS | YES | ND |
| Tinghir | H32/18 | 18 | NEG | NEG | NEG | YES | ND |
| Tinghir | H33/18 | 35 | POS | POS | NEG | NO |  |
| Tinghir | H34/18 | 68 | NEG | NEG | NEG | NO |  |
| Tinghir | H35/17 | 22 | NEG | NEG | NEG | YES | ND |
| Tinghir | H36/18 | 30 | NEG | NEG | NEG | YES | ND |
| Tinghir | H37/18 | 25 | NEG | NEG | NEG | YES | ND |
| Tinghir | H38/17 | - | POS | POS | NEG | NO |  |
| Tinghir | H39/18 | - | NEG | NEG | NEG | NO |  |
| Tinghir | H40/18 | 27 | POS | POS | POS | YES | ND |
| Tinghir | H41/18 | 33 | POS | POS | EQUIVOQUE | YES | ND |
| Tinghir | H42/18 | 35 | POS | POS | NEG | YES | ND |
| Tinghir | H43/18 | 45 | POS | POS | NEG | NO |  |
| Tinghir | H44/18 | 32 | NEG | NEG | NEG | YES | ND |
| Tinghir | H45/18 | 50 | POS | POS | NEG | NO |  |
| Tinghir | H46/18 | 19 | NEG | NEG | NEG | NO |  |
| Tinghir | H47/18 | 39 | POS | POS | NEG | NO |  |
| Tinghir | H48/18 | 52 | POS | POS | NEG | NO |  |
| Tinghir | H49/18 | 55 | POS | POS | NEG | NO |  |
| Tinghir | H50/18 | 45 | POS | POS | NEG | NO |  |
| Tinghir | H51/18 | 24 | POS | POS | NEG | NO |  |
| Tinghir | H52/18 | 36 | POS | POS | POS | NO |  |
| Tinghir | H53/18 | 28 | NEG | NEG | NEG | YES | ND |
| Tinghir | H54/18 | 23 | POS | POS | NEG | YES | ND |
| Tinghir | H55/18 | 22 | POS | POS | NEG | YES | ND |
| Tinghir | H56/18 | - | POS | POS | NEG | YES | ND |
| Tinghir | H57/18 | 26 | NEG | NEG | NEG | YES | ND |
| Tinghir | H58/18 | 52 | POS | POS | NEG | NO |  |
| Tinghir | H59/18 | 30 | NEG | NEG | NEG | YES | ND |
| Tinghir | H60/18 | 27 | POS | POS | NEG | YES | ND |
| Tinghir | H61/18 | - | NEG | POS | POS | NO |  |
| Tinghir | H62/18 | - | POS | POS | NEG | NO |  |
| Tinghir | H63/18 | 61 | NEG | NEG | NEG | NO |  |
| Tinghir | H64/18 | 21 | NEG | NEG | NEG | NO |  |
| Tinghir | H65/18 | 24 | NEG | NEG | NEG | YES | ND |
| Tinghir | H66/18 | 21 | POS | POS | NEG | YES | ND |
| Tinghir | H67/18 | 34 | NEG | NEG | NEG | NO |  |
| Tinghir | H68/18 | 27 | NEG | NEG | NEG | YES | ND |
| Tinghir | H69/17 | - | POS | POS | POS | YES | ND |
| Tinghir | H70/18 | 46 | POS | POS | NEG | NO |  |
| Tinghir | H71/18 | 52 | NEG | NEG | NEG | NO |  |
| Tinghir | H72/18 | 53 | NEG | NEG | NEG | NO |  |
| Tinghir | H73/18 | 25 | POS | POS | POS | YES | ND |
| Tinghir | H74/18 | 26 | NEG | NEG | NEG | NO |  |
| Tinghir | H75/18 | 24 | POS | POS | NEG | YES | ND |
| Tinghir | H76/18 | 26 | POS | POS | NEG | YES | ND |
| Tinghir | H77/18 | 37 | NEG | NEG | NEG | NO |  |
| Tinghir | H78/18 | 51 | NEG | NEG | NEG | NO |  |
| Tinghir | H79/18 | 58 | NEG | POS | NEG | NO |  |
| Tinghir | H80/18 | 27 | NEG | NEG | NEG | YES | ND |
| Tinghir | H81/18 | - | POS | POS | NEG | YES | ND |
| Tinghir | H82/18 | 50 | POS | POS | NEG | NO |  |
| Tinghir | H83/18 | 22 | POS | POS | EQUIVOQUE | NO |  |
| Tinghir | H84/18 | 23 | POS | POS | NEG | YES | ND |
| Tinghir | H85/18 | 26 | NEG | NEG | NEG | YES | ND |
| Tinghir | H86/18 | 36 | NEG | POS | NEG | YES | ND |
| Tinghir | H87/18 | 32 | NEG | NEG | NEG | YES | ND |
| Tinghir | H88/18 | 40 | POS | POS | EQUIVOQUE | YES | ND |
| Tinghir | H89/18 | 30 | POS | POS | POS | YES | ND |
| Tinghir | H90/18 | 24 | NEG | NEG | NEG | YES | ND |
| Tinghir | H91/18 | 32 | POS | POS | NEG | NO |  |
| Tinghir | H92/18 | 55 | POS | POS | NEG | NO |  |
| Tinghir | H93/18 | 70 | NEG | NEG | NEG | NO |  |
| Tinghir | H94/18 | 25 | NEG | POS | NEG | NO |  |
| Tinghir | H95/18 | 26 | POS | POS | NEG | NO |  |
| Tinghir | H96/18 | 37 | POS | POS | NEG | NO |  |
| Tinghir | H97/18 | 50 | NEG | NEG | NEG | NO |  |
| Tinghir | H98/18 | 23 | POS | POS | NEG | NO |  |
| Tinghir | H99/18 | 34 | NEG | NEG | NEG | NO |  |
| Tinghir | H100/18 | 45 | NEG | NEG | NEG | NO |  |
| Tinghir | H101/18 | 43 | NEG | NEG | NEG | NO |  |
| Tinghir | H102/18 | 53 | POS | POS | NEG | NO |  |
| Tinghir | H103/18 | 64 | POS | POS | NEG | NO |  |
| Tinghir | H104/18 | 65 | POS | POS | NEG | NO |  |
| Tinghir | H105/18 | 48 | POS | POS | NEG | NO |  |
| Tinghir | H106/18 | 59 | NEG | NEG | NEG | NO |  |
| Tinghir | H107/18 | 65 | NEG | POS | NEG | NO |  |
| Tinghir | H108/18 | 50 | NEG | NEG | NEG | NO |  |
| Tinghir | H109/18 | - | POS | POS | NEG | NO |  |
| Tinghir | H110/18 | 51 | NEG | NEG | NEG | NO |  |
| Tinghir | H111/18 | 51 | NEG | NEG | NEG | NO |  |
| Tinghir | H112/18 | 40 | NEG | NEG | NEG | NO |  |
| Tinghir | H113/18 | 69 | POS | ND | ND | NO |  |
| Tinghir | H114/18 | 55 | POS | ND | ND | NO |  |
| Tinghir | H115/18 | - | POS | POS | NEG | NO |  |
| Tinghir | H116/18 | 22 | NEG | NEG | ND | NO |  |
| Tinghir | H117/18 | 47 | NEG | NEG | NEG | NO |  |
| Tinghir | H118/18 | 26 | NEG | NEG | NEG | NO |  |
| Tinghir | H119/18 | 45 | NEG | ND | ND | NO |  |
| Tinghir | H120/18 | 50 | NEG | ND | ND | NO |  |
| Tinghir | H121/18 | 32 | NEG | ND | ND | NO |  |
